# Modified Systemic Inflammation Score Is an Independent Predictor of Long-Term Outcome in Patients Undergoing Surgery for Adenocarcinoma of the Esophagogastric Junction

**DOI:** 10.3389/fsurg.2021.622821

**Published:** 2021-11-08

**Authors:** Jianping Xiong, Wenzhe Kang, Fuhai Ma, Hao Liu, Shuai Ma, Yang Li, Peng Jin, Haitao Hu, Yantao Tian

**Affiliations:** Department of Pancreatic and Gastric Surgery, National Cancer Center/National Clinical Research Center for Cancer/Cancer Hospital, Chinese Academy of Medical Sciences, Peking Union Medical College, Beijing, China

**Keywords:** adenocarcinoma of the esophagogastric junction, modified systemic inflammation score, albumin, prognostic factors, lymphocyte-to-monocyte ratio

## Abstract

**Background:** The modified systemic inflammation score (mSIS), which is calculated by a composite score of the lymphocyte-to-monocyte ratio and the albumin content in serum, is identified as the new score to predict the prognosis for various cancers. However, its significance for patients with adenocarcinoma of esophagogastric junction (AEJ), who receive surgery, remains unclear.

**Methods:** This study retrospectively analyzed 317 patients with AEJ receiving surgery between September 2010 and December 2016. The associations between the mSIS and the clinicopathological features, overall survival (OS), as well as relapse-free survival (RFS), were assessed. In addition, the time-dependent receiver operating characteristic (t-ROC) curve analysis was performed for comparing the value of those scoring systems in predicting patient prognosis.

**Results:** Of the 317 cases, 119 were rated as mSIS 0, 123 as mSIS 1, and 75 as mSIS 2. Besides, mSIS was significantly related to age and tumor size. On multivariate analysis, mSIS was identified as a predictor to independently predict OS (*p* < 0.001) along with RFS (*p* < 0.001), and a significantly strong correlation was observed at the advanced pTNM stages based on the mSIS system. In the subgroup analysis of adjuvant chemotherapy and surgery alone, mSIS was still the predictor for independently predicting patient OS (*p* < 0.001) together with RFS (*p* < 0.001) for the two groups. T-ROC analysis showed that mSIS was more accurate than controlling nutritional status score in predicting OS and RFS.

**Conclusions:** The mSIS can serve as an easy, useful scoring system to independently predict the preoperative survival for AEJ cases undergoing surgery.

## Introduction

Adenocarcinoma of the esophagogastric junction (AEJ) is defined as the adenocarcinoma at the gastroesophageal junction with an epicenter in a range of 5 cm (1). The incidence of AEJ has increased rapidly over the last few decades, especially in western countries (2). Keeney et al. discovered that the incidence of AEJ increased by 20% in 1998; particularly, it elevated by three to four times in patients aged over 65 years (3). AEJ is an aggressive malignancy with 5-year survival after diagnosis of < 20% (4). The currently recognized prognostic factors for patients with AEJ include tumor stage, vascular invasion, and lymphatic invasion, but these postoperative factors cannot be used routinely, which has severely limited their clinical application. To more efficiently evaluate the risks of disease progression and postoperative death in patients with AEJ, we need to determine and characterize new factors.

Systemic inflammation exerts a vital part in cancer pathogenesis and occurrence; besides, the systemic inflammation markers are related to cancer survival (5, 6). The serum contents of inflammatory biomarkers before treatment, including the platelet-to-lymphocyte ratio (PLR), lymphocyte-to-monocyte ratio (LMR), as well as neutrophil-to-lymphocyte ratio (NLR), have been recognized to be related to the progression and prognosis of many cancers, like gastric cancer (GC), hepatocellular carcinoma (HCC), and esophageal cancer (EC) (7, 8). In addition, the preoperative serum albumin level has also been used to predict the survival outcomes for lung cancer (LC), GC and EC (9, 10). Recently, Galizia et al. have first reported that a new scoring system according to the LMR and albumin concentration in serum, which was called the modified systemic inflammation score (mSIS) and it might comprehensively reflect nutritional status and systemic inflammation in patients with cancer (11). The mSIS was strongly related to the survival for patients with GC, esophageal squamous cell carcinoma (ESCC), and lymphoma (12–14). Besides, it is suggested that, compared with other prognostic factors, including the modified Glasgow Prognostic Score and the original systemic inflammation score, the mSIS is more accurate in predicting survival (12, 13). Also, it is proposed that AEJ should be considered separately from EC or GC due to its unique clinicopathological characteristics and survival outcome (15). So far, the value of mSIS in predicting the prognosis for patients with AEJ after surgical resection has not been investigated. Therefore, the present retrospective cohort study was carried out at a large center to determine the significance of mSIS in predicting the prognosis for patients with AEJ and to investigate the associations between mSIS and other clinicopathological features.

## Materials and Methods

### Objects of Study

**Figure 1:**
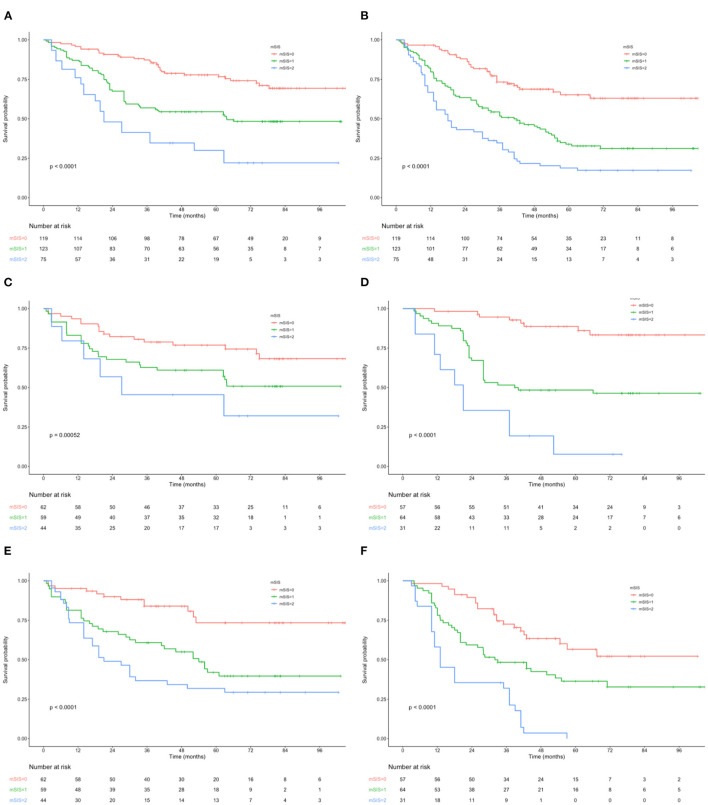
**(A)** Kaplan–Maier curves of OS for each mSIS group. **(B)** Kaplan–Maier curves of RFS for each mSIS group. **(C)** Association of the mSIS with the OS in the adjuvant chemotherapy group. **(D)** Association of the mSIS with the OS in the surgery-alone group. **(E)** Association of the mSIS with the RFS in the adjuvant chemotherapy group. **(F)** Association of the mSIS with the RFS in the surgery-alone group. OS, overall survival; RFS, relapse-free survival; AEG, adenocarcinoma of the gastroesophageal junction; mSIS, modified systemic inflammation score.

Adenocarcinoma of the esophagogastric junction cases receiving surgery from September 2010 to December 2016 at the Department of Pancreatic and Gastric Surgery in the National Cancer Center/Cancer Hospital, Chinese Academy of Medical Sciences, and Peking Union Medical College was evaluated in this study in a retrospective manner. The following inclusion criteria were applied: (1) a histologically confirmed adenocarcinoma of the esophagogastric junction; (2) no evidence of tumors invading adjacent organs, paraaortic lymph node enlargement, or distant metastasis demonstrated by abdominal computed tomography and/or magnetic resonance imaging (MRI) and posteroanterior chest radiography; and (3) a D1/D1 +/ D2 lymphadenectomy with a curative R0 resection. Patients conforming to any one of the criteria below were excluded from this study: (1) those receiving palliative surgery, (2) those who did not receive routine preoperative blood test, (3) those with distant metastasis (DM) at the time of surgery, (4) those receiving neoadjuvant chemotherapy (NCT), (5) those with other organ malignancies, (6) those receiving emergency operation, (7) those with other synchronous malignancies, (8) those with incomplete/inaccurate medical records, (9) those with chronic liver and/or kidney diseases, a chronic inflammatory disorder, active infection in any form, hematological diseases and autoimmune diseases, etc., (10) those with missing laboratory data, and (11) those with missing follow-up data. At last, 317 cases were included in the present study (Figure 1). The demographic, laboratory, and histopathological variables from the enrolled cases, together with the extracted data from our hospital database and patient records, were examined retrospectively. The clinical tumor stage was determined in accordance with the Pathological Tumor Lymph Node Metastasis (pTNM) classification (8th edition) released by the International Union for Cancer Control (UICC). Adenocarcinomas with epicenters are more than 2 cm from AEJ and enter the proximal stomach and are staged as stomach cancers; cardia cancer that does not involve AEJ (epicenters are < 2 cm from AEJ) is staged as stomach cancers; adenocarcinomas with epicenters no more than 2 cm into the gastric cardia are staged as esophageal adenocarcinomas. AEJs were classified into three subtypes (Type I, Type II, and Type III) according to Siewert's classification (16). The appropriate way of surgical procedure was selected according to the location of AEJ (abdominothoracic enbloc esophagectomy or transhiatal extended gastrectomy, respectively). The multidisciplinary team of oncology discussed the treatment for each patient before surgery. Oxaliplatin/capecitabine or cisplatin/5-fluorouracil was recommended for the patients with advanced AEJ as the adjuvant chemotherapy.

### Definition of Inflammation-Based Indicators

A routine blood test was performed a week before surgery. The results of blood tests conducted a week before surgery were obtained from the laboratory database of the National Cancer Center (Beijing, China). No patient developed the signs of pyrexia (axillary temperature ≥ 37.2°C/99.°CF), a chronic inflammatory disorder, or active infection in any form. Preoperative data, including body mass index (BMI), serum albumin concentration, tumor size, American Society of Anesthesiologists (ASA) score, total cholesterol level, and the absolute neutrophil/monocyte/lymphocyte counts, were collected. By using the method reported by Lin et al., mSIS was calculated by serum albumin content and LMR. For cases who had serum albumin concentration < 40 g/l and LMR < 3.4, the score was 2; for those having either serum albumin content ≥ 40 g/l or LMR ≥ 3.4, the score was 1; and for those having the serum albumin content ≥ 40 g/l and/or LMR ≥ 3.4, the score was 0 (Supplementary Table 1). The controlling nutritional status (CONUT) score was calculated according to the preoperative serum albumin level, total cholesterol content, and peripheral lymphocyte count (17) by the method reported previously (4) (Supplementary Table 2). MaxStat analysis was carried out to determine the optimal CONUT threshold. Patients were classified into high (≥3) and low (≤ 2) CONUT score groups.

### Follows-Up

Patients were followed up after surgery at intervals of 3 months within the first 2 years and intervals of 6 months thereafter. The last follow-up assessment was carried out in October 2019. The follow-up examinations included abdominopelvic CT, chest X-ray, tumor markers (including CEA, CA19-9, and CA72-4), and annual endoscopy. Overall survival (OS) was deemed as the primary endpoint, which indicated the duration between surgery and death due to all-cause or the final follow-up. Relapse-free survival (RFS) was regarded as the secondary endpoint, which represented the time between surgery and death or disease relapse. Death from any cause was considered an event.

### Statistical Methods

Categorical data were analyzed by chi-square test, while continuous data were analyzed by *t*-tests. Kaplan-Meier survival curve was plotted, and differences between the curves were analyzed using the log-rank test. Variables with *p* < 0.05 were identified based on univariate analysis, which were then incorporated into the Cox regression model for multivariate analysis. In addition, the time-dependent receiver operating characteristic (t-ROC) curves were plotted, while area under the curve (AUC) values were adopted for comparing mSIS and CONUT for their prognosis predicting performance (18). Each test was bilateral, and a difference of *p* < 0.05 was considered statistically significant. Rver.4.0.2 (R Foundation for Statistical Computing, Vienna, Austria) and SPSS18.0 (SPSS Inc., Chicago, IL, USA) were used for statistical analysis. Moreover, the “rms” function of the R package was used to calculate C-index values, while the “timeROC” function of the R package was employed for t-ROC analysis. The present study gained approval from the Ethics Review Committee of National Cancer Center/Cancer Hospital, Chinese Academy of Medical Sciences and Peking Union Medical College.

## Results

### Patient Characteristics

Totally, 317 AEJ cases were enrolled into the present study (Supplementary Figure 1). There were 265 (83.6%) male and 52 (16.7%) female patients. For all patients, their age at surgery ranged from 36 to 86 (average, 58.2) years. According to the pTNM classification system, there were 63 (19.9%) stage I patients, 93 (29.3%) stage II patients, and 161 (50.8%) stage III patients. Fifteen had type I (4.8%) AEJs, 190 had type II (59.9%), and 112 had type III (35.2%). Among the 317 patients, 165 (52%) underwent adjuvant chemotherapy. In line with the mSIS system, 119 patients (37.5%) were divided into the mSIS = 0 group, 123 (38.8%) were in the mSIS = 1 group, and 75 (23.7%) were in the mSIS = 2 groups (Table 1).

**Table 1:** Association of mSIS and clinicopathological characteristics in patients with AEJ.

**Clinicopathological features**	**All cases**	**mSIS0** **(***n*** = 119)**	**mSIS1** **(***n*** = 123)**	**mSIS2** **(***n*** = 75)**	***P*** **value**
Age (median)	63.4	59.5	63.9	68.4	0.003
GenderMaleFemale	265 (83.6) 52 (16.4)	94 (79.0)25 (21.0)	106 (85.4) 18 (14.6)	66 (87.0)9 (12.0)	0.112
BMI (median)	23.8	24.5	23.2	23.6	0.601
ASA score123	23 (7.3) 243 (76.7) 51 (16.0)	9 (7.5)93 (78.2)17 (14.3)	11 (8.9) 87 (70.8) 25 (20.3)	3 (4.0)63 (84.0)9 (12.0)	0.425
Tumor size (cm, median)	4.8	4.2	4.7	6.0	0.008
Tumor differentiationG1G2G3	36 (11.4) 180 (56.8) 101 (31.8)	13 (10.9)65 (54.7)41 (34.4)	16 (13.1) 79 (64.2) 28 (22.8)	7 (9.3)36 (48.0)32 (42.7)	0.185
Siewert classificationType IType IIType III	15 (4.8) 190 (59.9) 112 (35.3)	3 (2.5)43 (36.1)73 (61.3)	10 (8.1) 99 (80.4) 14 (11.5)	2 (2.7)48 (64.0)25 (33.3)	0.245
Vascular invasionNegativePositive	197 (62.1) 120 (37.9)	81 (68.1)38 (31.9)	75 (61.0) 48 (39.0)	39 (52.0)36 (48.0)	0.366
Perineural invasionNegativePositive	146 (66.1) 171 (53.9)	53 (44.6)66 (55.4)	59 (48.0) 64 (52.0)	34 (45.3)41 (54.7)	0.710
Lymphatic invasionNegativePositive	170 (53.7) 147 (46.3)	70 (58.5)49 (41.5)	63 (51.2) 60 (48.8)	37 (49.3)38 (50.7)	0.509
Surgical approachAbdominalThoracoabdominal	83 (24.2) 234 (73.8)	26 (21.8)93 (78.2)	45 (35.8) 78 (64.2)	12 (17.3)63 (82.7)	0.416
pTNM stageIIIIII	63 (19.9) 93 (29.3) 161 (50.8)	27 (22.7)39 (32.8)53 (44.5)	21 (17.1) 43 (35.9) 59 (48.0)	15 (20.0)11 (14.7)49 (65.3)	0.283
Adjuvant chemotherapyNoYes	152 (47.9) 165 (52.1)	57 (47.3)62 (52.1)	64 (52.1) 59 (47.9)	31 (41.3)44 (58.7)	0.609
Serum albumin (g/L)≥40 <40	180 (56.8) 137 (43.2)	115 (96.6)4 (3.4)	65 (52.8) 58 (48.2)	075 (100)	< 0.001
Lymphocyte: monocyte ratio≥3.4 <3.4	207 (65.3) 110 (34.9)	114 (97.8)5 (4.2)	93 (75.6) 30 (24.4)	075 (100)	< 0.001

*mSIS, modified systemic inflammation score; AEG, adenocarcinoma of the gastroesophageal junction; BMI, body mass index; ASA, American Society of Anesthesiologists*.

### Relationships of the Preoperative mSIS System With Clinicopathological Features

Table 1 presents the relationships of the mSIS system with clinicopathological features. As observed from the table, the mSIS was significantly related to age and tumor size. However, there was no difference in BMI, ASA score, tumor differentiation, perineural invasion, vascular involvement, pTNM stage, surgical approach, or adjuvant chemotherapy among the three mSIS groups. Moreover, mSIS increased among cases who had serum albumin (mg/dL) < 40 and LMR ≤ 3.40.

### OS and RFS Based on MSIS

**Figure 2:**
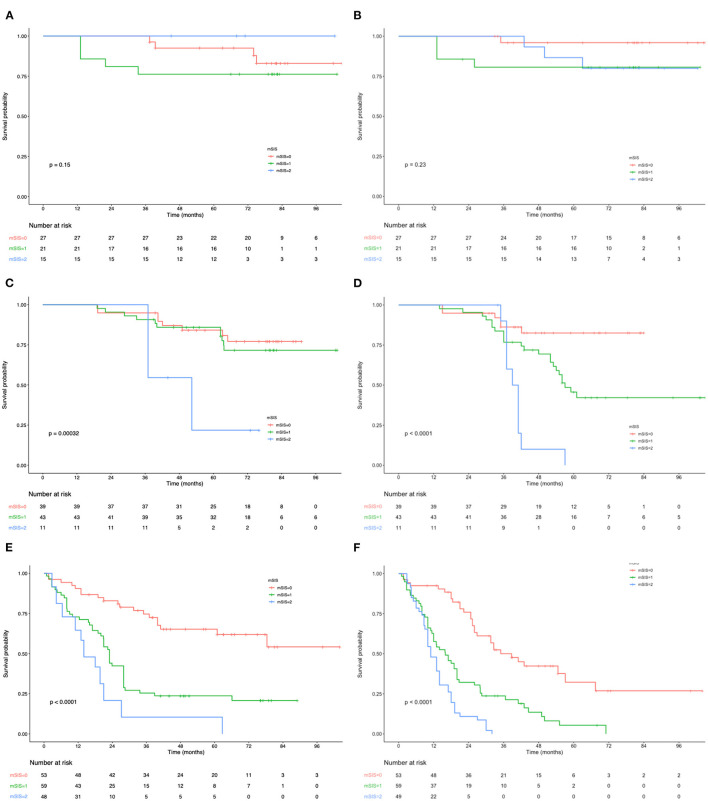
**(A)** Relationship between mSIS and the OS of patients with stage I AEJ. **(B)** Relationship between mSIS and the RFS of patients with stage I AEJ. **(C)** Relationship between mSIS and the OS of patients with stage II AEJ. **(D)** Relationship between mSIS and the RFS of patients with stage II AEJ. **(E)** Relationship between mSIS and the OS of patients with stage III AEJ. **(F)** Relationship between mSIS and the RFS of patients with stage III AEJ. OS, overall survival. RFS, relapse-free survival. AEG, adenocarcinoma of the gastroesophageal junction. mSIS, modified systemic inflammation score.

The OS and RFS curves were statistically analyzed, as depicted in Figure 1. The OS rates at 1, 3, and 5 years of the included cases were 87.6, 62.8, and 44.7%, respectively. The median OS and RFS of all included patients were 49.3 and 40.7 months, respectively. For the mSIS groups, the median OS of the mSIS = 0, 1, and 2 groups were 60.2, 48.3, and 33.7 months, respectively. Additionally, the median RFS of the mSIS = 0, 1, and 2 groups were 48.1, 40.4, and 29.3 months, respectively. The Kaplan–Meier survival analysis indicated that the high mSIS was related to the poor OS and RFS for all the included patients (Figures 1A,B). Furthermore, the survival differed significantly based on the mSIS in the surgery-alone group and the postoperative adjuvant chemotherapy group (Figures 1C–F). Moreover, after stratification according to the pTNM stage, the stage III subgroup showed the most significant differences in OS and RFS based on the mSIS system (Figure 2).

### Univariate and Multivariate Analysis on the Predictors for AEJ Patient Prognosis

On the one hand, univariate analysis found that tumor size (*HR* = 1.57, *p* < 0.001), tumor differentiation (G2: *HR* = 1.40, *P* = 0.009; G3: *HR* = 1.98, *P* = 0.004), pTNM stage (II:*HR* = 2.48, *P* = 0.002; III: *HR* = 6.10, *P* = 0.001), perineural invasion (*HR* = 2.02, *P* = 0.010), lymphatic invasion (*HR* = 2.63, *P* = 0.017), and vascular invasion (*HR* = 1.65, *p* < 0.001) were associated with OS (Table 2). In multivariate analyses, pTNM stage, vascular invasion, lymphatic invasion, and CONUT scores were the factors that independently predicted OS (Table 2; Supplementary Figure 2). On the other hand, univariate analysis determined that tumor differentiation, tumor size, pTNM stage, vascular invasion, lymphatic invasion, perineural invasion, and CONUT scores were the important prognostic factors (Table 3). Results of multivariate analysis suggested that perineural invasion (*HR* = 1.90, *p* < 0.001), vascular invasion (*HR* = 1.76, *p* < 0.001), and pTNM stage (II:*HR* = 1.80, *p* < 0.001; III: *HR* = 4.89, *p* < 0.001), together with CONUT scores (*HR* = 1.86, *p* < 0.001), were the factors to independently predict RFS (Table 3; Supplementary Figure 3). And we have analyzed the data stratified by the subgroup of AEJ (Supplementary Tables 3, 4).

**Table 2:** Univariate and multivariate analysis of clinicopathologic variables in relation to OS in patients with AEJ.

**Clinicopathological features**	**Univariate analysis**	***P*** **value**	**Multivariate analysis**	***P*** **value**
Age	1.44 (0.91, 2.15)	0.106		
Gender	Reference			
Male Female	0.85 (0.62–1.73)	0.381		
BMI	1.28 (0.72, 2.39)	0.210		
ASA score	Reference			
1 2 3	1.39 (0.64, 1.87) 1.23 (0.70, 2.18)	0.332 0.209		
Tumor size (cm)	1.57 (1.13, 2.21)	<0.001	1.46 (0.75, 1.89)	0.170
Tumor differentiation	Reference		Reference	
G1 G2 G3	1.40 (1.25, 2.42) 1.98 (1.37, 3.66)	0.009 0.004	1.32 (0.31, 2.67) 1.75 (0.53, 3.70)	0.213 0.310
Siewert classification	Reference		Reference	
Type I Type II Type III	0.79 (0.41, 1.48) 0.69 (0.34, 1.51)	0.124 0.402		
Vascular invasion	Reference		Reference	
Negative Positive	1.65 (1.23–2.10)	<0.001	1.49 (1.21–2.08)	0.001
Perineural invasion	Reference		Reference	
Negative Positive	2.02 (1.43–3.56)	0.010	1.85 (0.70–2.63)	0.430
Lymphatic invasion	Reference		Reference	
Negative Positive	2.63 (1.57–4.81)	0.017	2.10 (1.46–3.29)	0.001
Surgical approach	Reference		Reference	
Abdominal Thoracoabdominal	1.78 (0.91, 2.64)	0.091		
pTNM stage	Reference		Reference	
I II III	2.48 (1.67–4.36) 6.10 (2.79, 8.91)	0.002 0.001	1.90 (1.55–3.23) 4.65 (2.31, 6.28)	<0.001 <0.001
Adjuvant chemotherapy				
No Yes	Reference 1.12 (0.85, 1.47)	0.319	Reference	
COUNT scores	Reference		Reference	
Low (<2) High (≥3)	1.65 (1.16, 2.52)	0.002	1.49 (1.33, 2.18)	0.027
mSIS	Reference		Reference	
0 1 2	1.86 (1.20, 2.73) 2.84 (1.33, 3.27)	<0.001 <0.001	1.59 (1.14, 2.35) 2.26 (1.26, 2.79)	<0.001 <0.001

*OS, overall survival; AEG, adenocarcinoma of the gastroesophageal junction; BMI, body mass index; ASA, American Society of Anesthesiologists; COUNT, controlling nutritional status; mSIS, modified systemic inflammation score*.

**Table 3:** Univariate and multivariate analysis of clinicopathologic variables in relation to RFS in patients with AEJ.

**Clinicopathological features**	**Univariate analysis**		**Multivariate analysis**	
Age	1.15 (0.74, 1.43)	0.412		
Gender				
Male Female	Reference 0.76 (0.43–1.39)	0.581		
BMI	0.86 (0.52, 1.31)	0.205		
ASA score				
1 2 3	Reference 1.51 (0.63, 2.04) 1.42 (0.70, 2.13)	0.282 0.253		
Tumor size (cm)	1.33 (1.10, 2.34)	0.010	1.28 (0.62, 1.75)	0.201
Tumor differentiation				
G1 G2 G3	Reference 1.51 (1.16, 2.18) 2.30 (1.55, 3.49)	0.022 0.005	Reference 1.32 (0.89, 1.67) 1.89 (0.93, 2.96)	0.113 0.085
Siewert classification				
Type I Type II Type III	Reference 0.73 (0.35, 1.42) 0.61 (0.40, 1.29)	0.107 0.518		
Vascular invasion				
Negative Positive	Reference 2.01 (1.45–3.22)	0.002	Reference 1.76 (1.23–2.45)	<0.001
Perineural invasion				
Negative Positive	Reference 2.37 (1.42–4.88)	0.009	Reference 1.90 (1.37–3.25)	<0.001
Lymphatic invasion				
Negative Positive	Reference 3.29 (1.62–5.74)	0.021	Reference 1.85 (1.31–3.02)	<0.001
Surgical approach				
Abdominal Thoracoabdominal	1.63 (0.93, 2.11)	0.101		
pTNM stage				
I II III	Reference 2.01 (1.35–4.62) 7.38 (3.67, 9.83)	0.003 0.009	Reference 1.76 (1.22–3.14) 4.89 (2.76, 6.83)	<0.001 <0.001
Adjuvant chemotherapy				
No Yes	1.18 (0.73, 1.55)	0.317		
COUNT scores				
Low (<2) High (≥3)	Reference 2.32 (1.47, 4.96)	0.014	Reference 1.86 (1.33, 2.25)	<0.001
mSIS				
0 1 2	Reference 2.41 (1.52, 5.33) 3.02(1.64, 6.40)	0.008 0.017	Reference 1.98 (1.40, 2.66) 2.15 (1.58, 4.51)	<0.001 <0.001

*RFS, relapse-free survival; AEG, adenocarcinoma of the gastroesophageal junction; BMI, body mass index; ASA, American Society of Anesthesiologists; COUNT, controlling nutritional status; mSIS, modified systemic inflammation score*.

### Prognostic Value of mSIS

**Figure 3:**
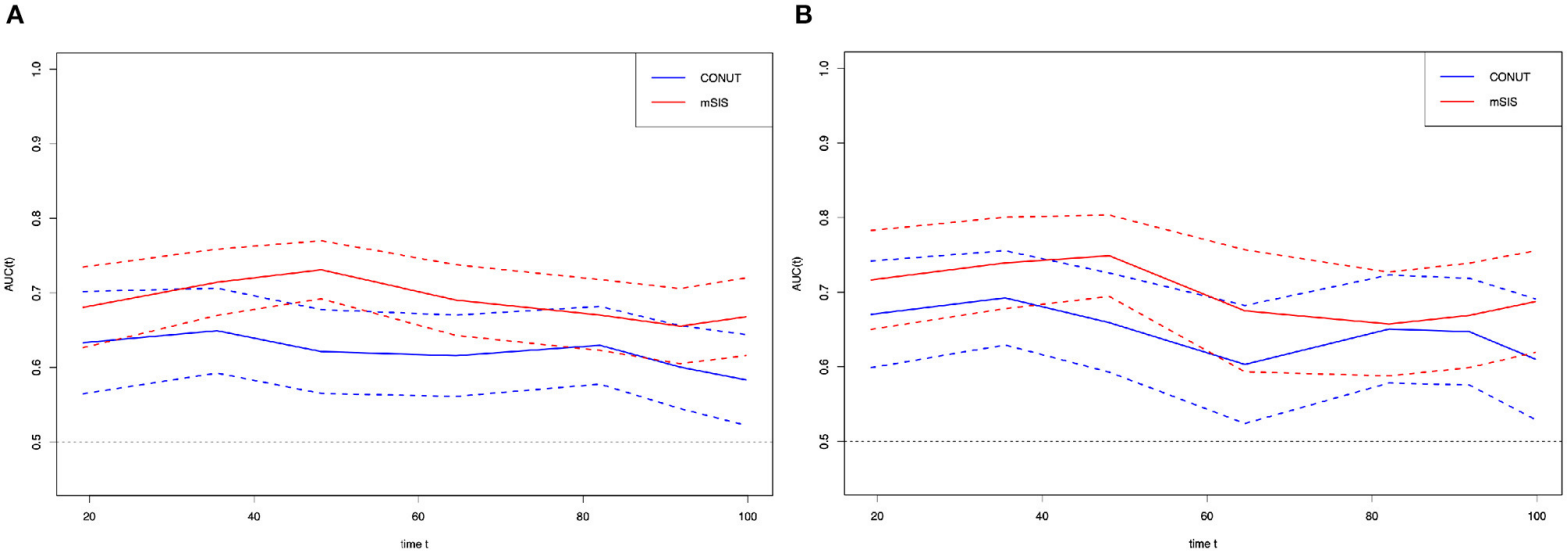
Time-dependent ROC curves for the mSIS and CONUT. The horizontal axis represents the year after surgery, and the vertical axis represents the estimated AUC for survival at the time of interest. Red and blue solid lines represent the estimated AUCs for the mSIS and CONUT, respectively, and broken lines represent the 95% confidence intervals for each AUC. **(A)** overall survival. **(B)** relapse-free survival. CONUT, controlling nutritional status. mSIS, modified systemic inflammation score; AUC, area under the curve; ROC, receiver operating characteristic.

Time-dependent receiver operating characteristic curve was plotted to compare the prognostic values of mSIS and CONUT (Figure 3). During the whole observation period, the t-ROC curve for mSIS still outperformed that for CONUT. Furthermore, when evaluating the predicting performance of mSIS and CONUT for OS, at 3 and 5 years postoperatively, the values of AUC for CONUT remarkably decreased compared with those for mSIS (3-year: 0.658 vs. 0.709, *P* = 0.010; 5 years: 0.616 vs. 0.701, *p* < 0.001). Also, the mSIS showed significantly higher accuracy than CONUT in predicting the 5-year RFS (5 years: 0.695 vs. 0.620, *p* < 0.001). In addition, the time-dependent receiver operating characteristic (t-ROC) curves and the predicted values of area under the curve (AUC) were also used to compare the prognostic value of mSIS, ALB, and LMR. As suggested by the results of t-ROC curve analysis to predict OS by different scoring systems, the AUC value was high for mSIS compared with those for other scoring systems. Typically, the AUC values in the prediction of 5-year OS were 0.695, 0.519, and 0.491 for mSIS, ALB, and LMR, respectively.

## Discussion

The present study had first assessed the associations between preoperative mSIS, clinicopathological factors, and survival, and investigated the significance of mSIS in predicting prognosis for AEJ cases who received surgery. The results indicated that mSIS was strongly associated with age and tumor size. The higher mSIS showed a tight correlation with the shorter survival, as indicated by univariate analysis. More importantly, mSIS was recognized to be a factor that independently predicted survival, and an especially strong correlation was observed at advanced pTNM stages. Besides, t-ROC curves were plotted for comparing mSIS and CONUT for their prognosis-predicting performance. Our study showed that mSIS tended to be superior to CONUT in predicting the 3- and 5-year OS along with the 5-year RFS.

Since Virchow first systematically reported the relationship of inflammation with cancer in the 19th century, an increasing number of studies have shown that systemic inflammation is an important part of the tumor microenvironment (TME) (5, 19). Accumulating evidence indicates that the inflammatory reaction in the microenvironment contributes to tumor progression including the induction of angiogenesis, tumor cell proliferation, and metastasis (20). As identified by accumulating studies, the inflammation-related prognosis scoring systems, including NLR, PLR, or LMR, are related to cancer prognoses, like GC, HCC, or ESCC (21, 22). However, the situation of the host can affect the prognostic abilities of a single inflammation-related marker, whereas a single marker may even be misleading when the cutoff value is arbitrarily determined. Recently, many studies have reported that mSIS, which is determined according to the LMR and serum albumin concentration before surgery, is the new inflammation-related prognosis scoring system. Researchers have identified that mSIS is of prognostic value for GC, lymphoma, and ESCC (12–14). It takes into account the effects of systemic inflammation together with the nutritional status on cancer prognosis. As a result, mSIS outperforms other single inflammatory or nutritional markers. As suggested by the results of t-ROC curve analysis to predict OS by different scoring systems, the AUC value was high for mSIS compared with those for ALB and LMR. Thus, mSIS was more accurate than its single components (ALB and LMR) in predicting OS.

Growing studies have determined the relationships of systemic inflammation and malnutrition with tumorgenesis, growth, metastasis, and progression. This has been confirmed in various cancers, including GC and EC; as a result, more efforts have been devoted to looking for the inflammation- and nutrition-related markers and developing a novel prognostic scoring system. In particular, the reduced albumin content in serum can serve as a sign of malnutrition and systemic inflammation. Tserum albumin content may be reduced by pro-inflammatory factors, including interleukin 6 (IL-6) and tumor necrosis factor-alpha (TNF-a), which directly inhibit hepatocyte albumin production (23, 24). The serum albumin concentration is currently included in most scoring systems. Hyperproteinemia is related to the better survival of AEJ, consistent with our results. LMR is linked with the numbers of monocytes and lymphocytes. Of them, lymphocytes are the fundamental unit in the innate and adaptive immune systems, which also lay the cell foundation for immune surveillance as well as immune editing (25). Lymphocytes can enhance the cancer immune surveillance ability, thereby inhibiting the proliferation, invasion, and metastasis of tumor cells (25). It is indicated that tumor-infiltrating lymphocytes are associated with the improved prognosis for various cancers, which may be attributed to the anti-tumor activity and angiogenesis inhibition induced by tumor-infiltrating lymphocytes (26). Therefore, lymphopenia is associated with a poor prognosis for patients with cancer. Previous studies have reported that the monocytes in circulation possibly facilitate cancer growth while decreasing immune monitoring (27). In addition, studies have shown that monocytes may promote the metastasis of tumor cells through tumor-monocyte-endothelial interaction (28). The increased number of neutrophils around the tumor may inhibit cytotoxic CD8 T cells for their anti-tumor activity, resulting in tumor growth and metastasis (29). Therefore, the increased number of monocytes predicts the poor prognosis for cancer patients, which indirectly indicates the relationship between low LMR and poor prognosis. Consistent with previous studies, LMR was found to exert a vital part in AEJ prognosis in this study. Additionally, as revealed by multivariate analysis, LMR might independently predict OS and RFS.

The CONUT score, which is determined according to the preoperative albumin level in serum, total cholesterol level, and peripheral lymphocyte count, is related to cancer survival and has been proved as the robust inflammatory-related score (17, 30, and 31). It is also considered a useful prognostic indicator for the long-term outcomes of GC and ESCC (30, 31). As suggested by the results of our study, CONUT was associated with AEJ prognosis, which was found to independently predict OS and RFS. Besides, this study compared the prognostic values of mSIS with CONUT. The t-ROC curve analysis using mSIS and CONUT was performed to predict OS and RFS, which indicated a trend that mSIS had higher AUC than CONUT, indicating that mSIS showed remarkably higher accuracy than SIS in predicting patient survival.

Compared with the existing tools that target immunonutritional interventions, our system had the major strength that, by combining oncological, nutritional, and immunological parameters, it outperformed the existing nutritional indices for predicting the survival outcome, and it targeted the immunonutritional intervention for candidate patients who might benefit the most. The results of our study indicated that early inflammation control and nutritional support might improve the prognosis for patients with cancer. Additionally, mSIS can be identified preoperatively; therefore, it can be used to facilitate decision-making for treatment before surgery and better estimate the survival outcome after surgery. Preoperative identification of patient status could have several uses in clinical practice, including prognostic stratification and treatment. Early detection and improvement of malnutrition and inflammation may result in better patient outcomes (32). Currently, we are investigating this tool in our patients with AEJ to preoperatively improve immune status in patients who have an mSIS score of 1 or 2. In addition, the use of anti-inflammatory agents, such as aspirin or other non-steroidal anti-inflammatory drugs, has been shown to attenuate systemic inflammation and cachexia, improve the postoperative course, and ameliorate tolerance of anticancer therapy and long-term outcome (32–34). Preoperative mSIS is a simple, easily obtainable scoring system strongly associated with an outcome in patients with AEJ who are undergoing surgery. Meanwhile, patients with an elevated preoperative mSIS should be considered at high risk of tumor relapse and considered for tailored therapy. Indeed, our multidisciplinary team is now considering administering adjuvant chemotherapy to patients with early AEJ and a high mSIS, who are undergoing potentially curative surgery. Additional studies will determine whether this strategy will turn out to be rewarding.

Certain limitations should be noted in this study. The main limitation of this study is its lack of neoadjuvated patients and retrospective design. First, although our study strictly complied with the inclusion and exclusion criteria, there was still selection bias due to its retrospective nature. In addition, patients were included from just one institution, and the cohort was ethnically homogeneous. Second, patients who received neoadjuvant were excluded in order to avoid substantial variations in peripheral blood components induced from the anticancer therapies before surgery, which might lead to confounding bias risks that complicate the actual impact of NPS on postoperative survival. Despite that patients who received neoadjuvant therapy were excluded strictly, we were not sure whether each case was under identical status prior to blood sample collection. Besides, our findings did not apply to AEJ cases receiving neoadjuvant therapy.

## Conclusions

According to results in the present study, preoperative mSIS can serve as a simple and useful predictor to predict AEJ prognosis. Furthermore, it may be adopted to be one part of preoperative prognosis classification and postoperative follow-up for developing an individual treatment for AEJ cases.

## Data Availability Statement

The original contributions presented in the study are included in the article/Supplementary Material, further inquiries can be directed to the corresponding author/s.

## Ethics Statement

The studies involving human participants were reviewed and approved by Ethics Review Committee of National Cancer Center/Cancer Hospital, Chinese Academy of Medical Sciences and Peking Union Medical College. The patients/participants provided their written informed consent to participate in this study.

## Author Contributions

JX conceived the study and wrote the manuscript. WK, FM, and HH searched the database, reviewed the studies, and collected the data. HL and SM performed the statistical analyses. YL and PJ performed revision of the manuscript. YT arranged for and provided the funding for this study. All the authors reviewed the manuscript and participated in its revision. YT is the guarantor for this study. All authors contributed to the article and approved the submitted version.

## Funding

This study was supported by grants from the National Natural Science Foundation of China (81772642), the Beijing Municipal Science & Technology Commission (Z161100000116045), and Capital's Funds for Health Improvement and Research (CFH 2018-2-4022). The funders had no role in study design, data collection, and analysis, decision to publish, or preparation of the manuscript.

## Conflict of Interest

The authors declare that the research was conducted in the absence of any commercial or financial relationships that could be construed as a potential conflict of interest.

## Publisher's Note

All claims expressed in this article are solely those of the authors and do not necessarily represent those of their affiliated organizations, or those of the publisher, the editors and the reviewers. Any product that may be evaluated in this article, or claim that may be made by its manufacturer, is not guaranteed or endorsed by the publisher.

## Supplementary Material

Supplementary Figure 1 A flow diagram of the cases analyzed. AEG, adenocarcinoma of the gastroesophageal junction.

Supplementary Figure 2 A, overall survival curves according to the preoperative ALB. B, relapse-free survival curves according to the preoperative ALB. C, overall survival curves according to the preoperative LMR. D, relapse-free survival curves according to the preoperative LMR. ALB, albumin; LMR, lymphocyte-to-monocyte ratio.

Supplementary Figure 3 A Kaplan-Maier curves of overall survival in the CONUT-high (≥ 3) and CONUT-low (≤ 2) score groups. B, Kaplan-Maier curves of relapse-free survival in the CONUT-high (≥ 3) and CONUT-low (≤ 2) score groups. CONUT, controlling nutritional status.

Supplementary Table 1 The definition of modified mSIS. mSIS, modified systemic inflammation score; ALB, albumin; LMR, lymphocyte-to-monocyte ratio.

Supplementary Table 2 The definition of CONUT. CONUT, controlling nutritional status; ALB, albumin; TLC, total lymphocyte; TC, total cholesterol.

Supplementary Table 3 Univariate and multivariate analysis of clinicopathologic variables in relation to OS in patients with AEJ. OS, and overall survival. AEG, adenocarcinoma of the gastroesophageal junction; BMI, body mass index; ASA, American Society of Anesthesiologists; COUNT, controlling nutritional status; mSIS, modified systemic inflammation score.

Supplementary Table 4 Univariate and multivariate analysis of clinicopathologic variables in relation to RFS in patients with AEJ. RFS, relapse-free survival; AEG, adenocarcinoma of the gastroesophageal junction; BMI, body mass index; ASA, American Society of Anesthesiologists; COUNT, controlling nutritional status; mSIS, modified systemic inflammation score.
