# Supplementary figures and images for: Modified Systemic Inflammation Score Is an Independent Predictor of Long-Term Outcome in Patients Undergoing Surgery for Adenocarcinoma of the Esophagogastric Junction
Source: Front Surg. 2021 Nov 8;8:622821. doi: 10.3389/fsurg.2021.622821 (PMC8606684; doi:10.3389/fsurg.2021.622821)

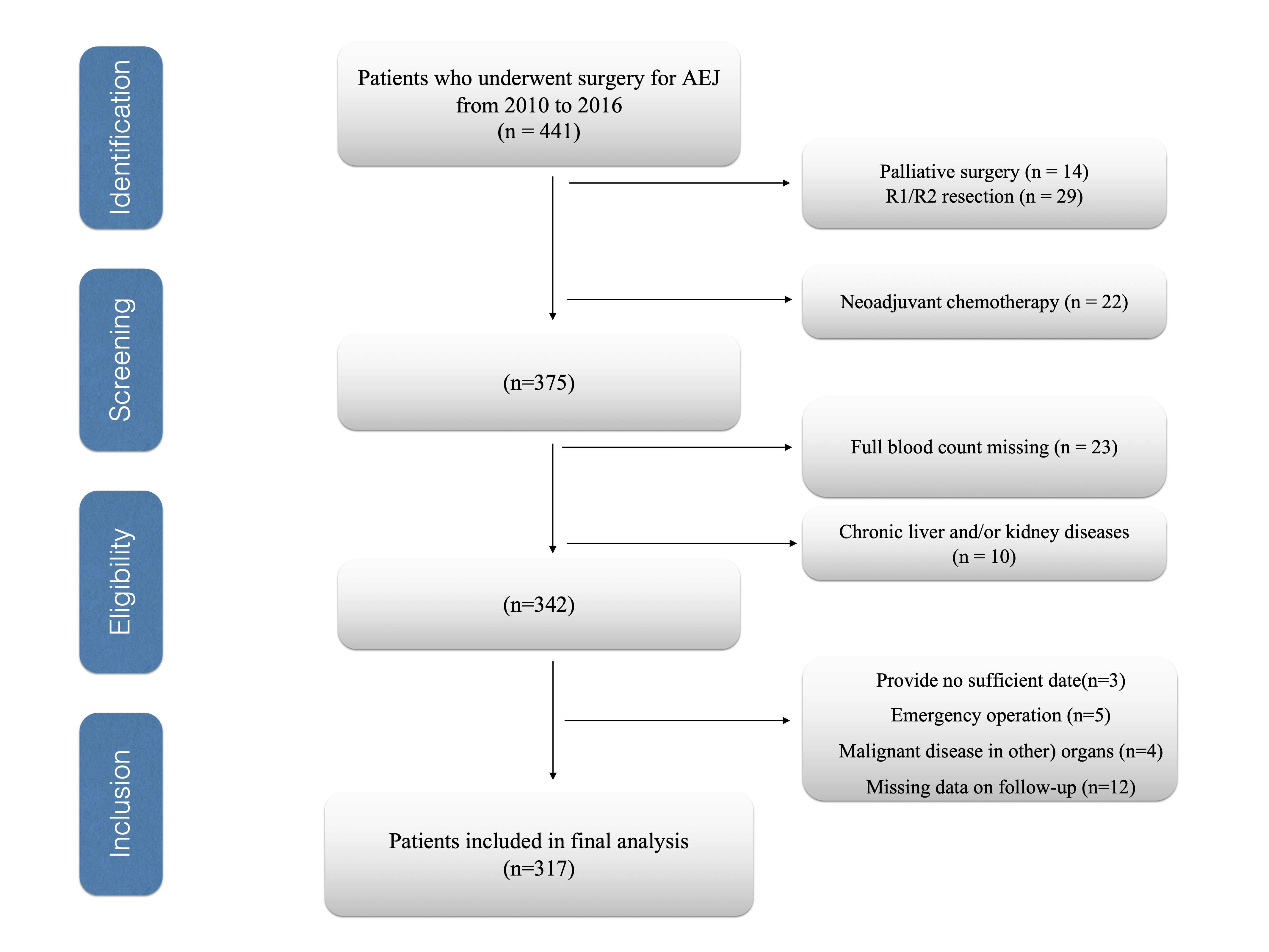

Supplement: Supplementary Figure 1 — A flow diagram of the cases analyzed. AEG, adenocarcinoma of the gastroesophageal junction. [file Image_1.TIFF]

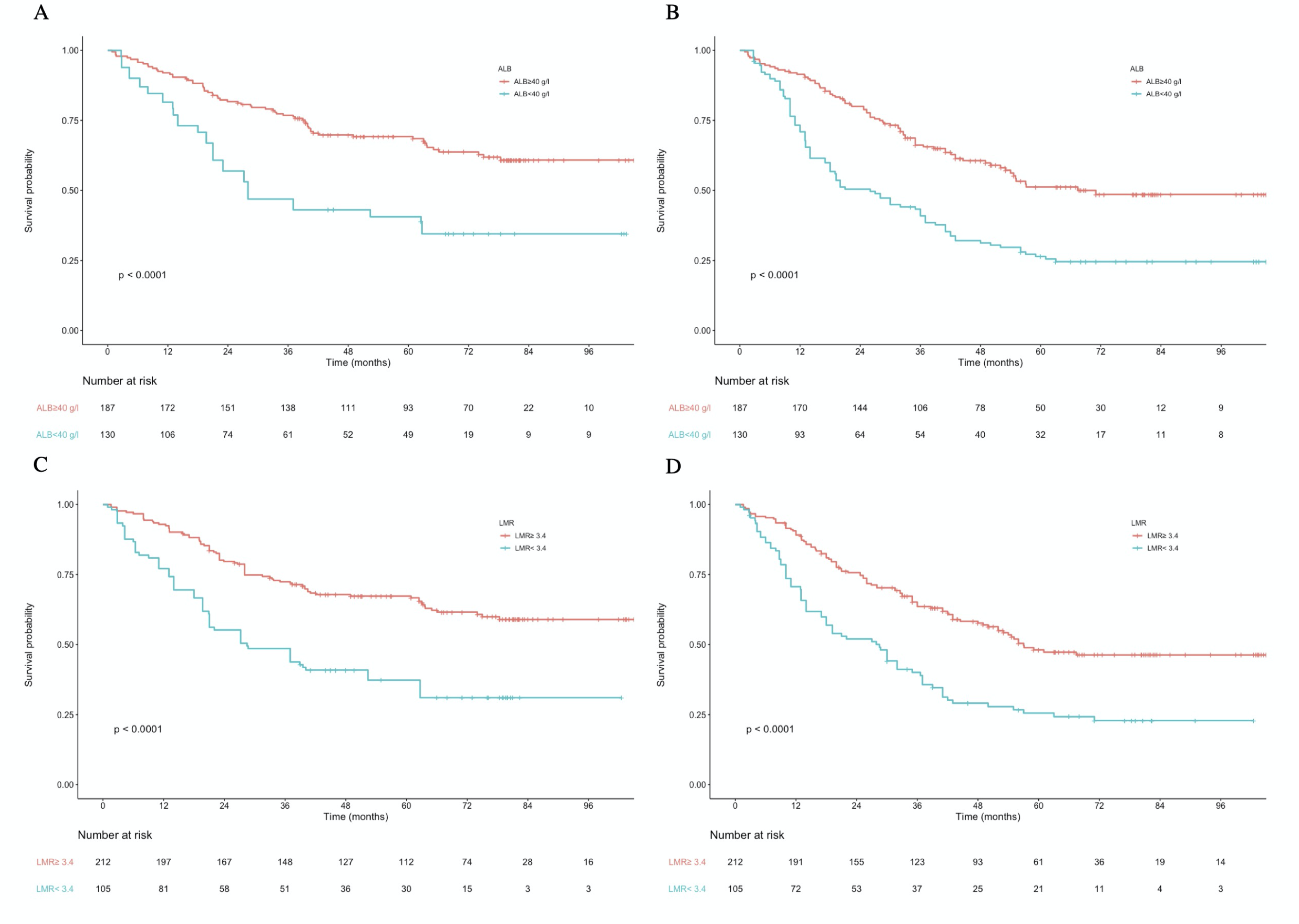

Supplement: Supplementary Figure 2 — A, overall survival curves according to the preoperative ALB. B, relapse-free survival curves according to the preoperative ALB. C, overall survival curves according to the preoperative LMR. D, relapse-free survival curves according to the preoperative LMR. ALB, albumin; LMR, lymphocyte-to-monocyte ratio. [file Image_2.TIFF]

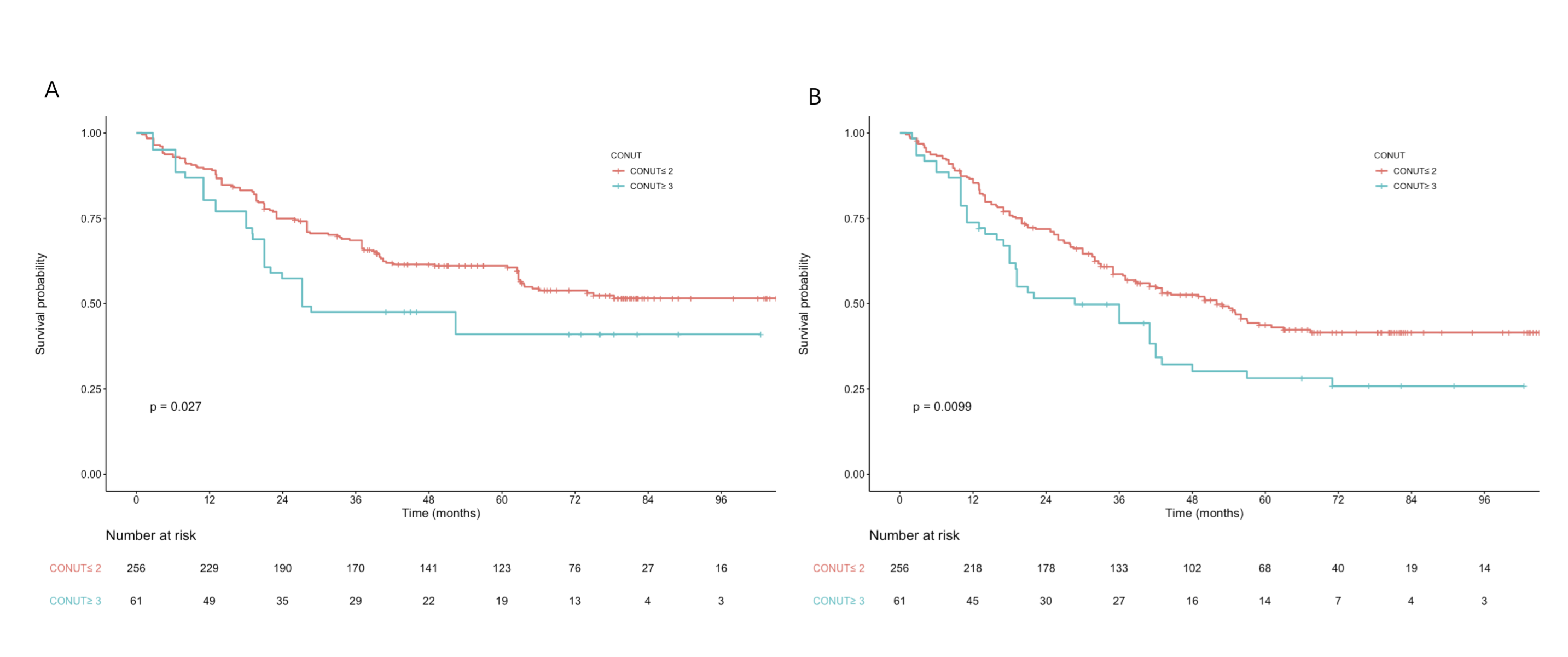

Supplement: Supplementary Figure 3 — A Kaplan-Maier curves of overall survival in the CONUT-high (≥ 3) and CONUT-low (≤ 2) score groups. B, Kaplan-Maier curves of relapse-free survival in the CONUT-high (≥ 3) and CONUT-low (≤ 2) score groups. CONUT, controlling nutritional status. [file Image_3.TIFF]
