# Supplementary material for: Modified Systemic Inflammation Score Is an Independent Predictor of Long-Term Outcome in Patients Undergoing Surgery for Adenocarcinoma of the Esophagogastric Junction
Source: Front Surg. 2021 Nov 8;8:622821. doi: 10.3389/fsurg.2021.622821 (PMC8606684; doi:10.3389/fsurg.2021.622821)
Supplement: Supplementary Table 1 — The definition of modified mSIS. mSIS, modified systemic inflammation score; ALB, albumin; LMR, lymphocyte-to-monocyte ratio. [file Table_1.DOCX]

| **Scoring System** | **Score** |
| --- | --- |
| The mSIS |  |
| Alb (≥40 g/l ) and LMR (≥3.40) | 0 |
| Alb (≥40 g/l ) and LMR (<3.40) | 0 |
| Alb (<40 g/l ) and LMR (≥3.40 ) | 1 |
| Alb (<40 g/l ) and LMR (<3.40 ) | 2 |

**Supplemental Table 1**. The definition of modified mSIS. mSIS, modified systemic inflammation score. ALB, albumin. LMR, lymphocyte-to-monocyte ratio.
