# Supplementary material for: Modified Systemic Inflammation Score Is an Independent Predictor of Long-Term Outcome in Patients Undergoing Surgery for Adenocarcinoma of the Esophagogastric Junction
Source: Front Surg. 2021 Nov 8;8:622821. doi: 10.3389/fsurg.2021.622821 (PMC8606684; doi:10.3389/fsurg.2021.622821)
Supplement: Supplementary Table 2 — The definition of CONUT. CONUT, controlling nutritional status; ALB, albumin; TLC, total lymphocyte; TC, total cholesterol. [file Table_2.DOCX]

| **Parameters** | **CONUT** | | | |
| --- | --- | --- | --- | --- |
| Serum albumin (g/dL) | 3.5–4.5 | 3.0–3.49 | 2.5–2.9 | <2.5 |
| ALB score | 1 | 2 | 4 | 6 |
| Total lymphocyte (count/mm3) | ≥ 1600 | 1200–1599 | 800-1199 | <800 |
| TLC score | 0 | 1 | 2 | 3 |
| Total cholesterol (mg/dL) | >180 | 140-180 | 100-139 | <100 |
| TC score | 0 | 1 | 2 | 3 |
| CONUT score (total) | 0-1 | 2-4 | 5-8 | 9-12 |
| Assessment | Normal | Light | Moderate | Severe |

**Supplemental Table 2.** The definition of CONUT. CONUT, controlling nutritional status. ALB, albumin. TLC, Total lymphocyte. TC, Total cholesterol.
