# Supplementary material for: Modified Systemic Inflammation Score Is an Independent Predictor of Long-Term Outcome in Patients Undergoing Surgery for Adenocarcinoma of the Esophagogastric Junction
Source: Front Surg. 2021 Nov 8;8:622821. doi: 10.3389/fsurg.2021.622821 (PMC8606684; doi:10.3389/fsurg.2021.622821)
Supplement: Supplementary Table 3 — Univariate and multivariate analysis of clinicopathologic variables in relation to OS in patients with AEJ. OS, and overall survival. AEG, adenocarcinoma of the gastroesophageal junction; BMI, body mass index; ASA, American Society of Anesthesiologists; COUNT, controlling nutritional status; mSIS, modified systemic inflammation score. [file Table_3.DOCX]

| Siewert classification | Type I | | Type II | | Type III | |
| --- | --- | --- | --- | --- | --- | --- |
| Clinicopathological features | HR (95% CI) | P value | HR (95% CI) | P value | HR (95% CI) | P value |
| Age | 1.42 (0.95, 2.19) | 0.102 | 1.43 (0.98, 2.11) | 0.102 | 1.48 (0.98, 2.42) | 0.111 |
| Gender  Male  Female | Reference  0.68(0.48–1.98) | 0.392 | Reference  0.81 (0.61–1.72) | 0.380 | Reference  0.95 (0.69–1.79) | 0.399 |
| BMI | 1.21 (0.71, 2.40) | 0.211 | 1.26 (0.78, 2.45) | 0.218 | 1.32 (0.81, 2.48) | 0.209 |
| ASA score  1  2  3 | Reference  1.28 (0.48, 1.98)  1.22 (0.67, 2.01) | 0.323  0.222 | Reference  1.36 (0.62, 1.89)  1.29 (0.72, 2.09) | 0.176  0.357 | Reference  1.54 (0.69. 2.02)  1.20 (0.67, 2.32) | 0.381  0.267 |
| Tumor size (cm) | 1.54 (1.12, 2.12) | < 0.001 | 1.51 (1.12, 2.22) | < 0.001 | 1.59 (1.12, 2.23) | < 0.001 |
| Tumor differentiation  G1  G2  G3 | Reference  1.37 (1.22, 2.40)  1.90 (1.21, 3.41) | 0.005  0.002 | Reference  1.49 (1.25, 2.46)  1.76 (1.30, 3.61) | 0.007  0.009 | Reference  1.40 (1.25, 2.45)  2.12 (1.67, 3.90) | 0.010  0.008 |
| Vascular invasion  Negative  Positive | Reference  1.61 (1.22–2.08) | < 0.001 | Reference  1.60 (1.20–2.01) | < 0.001 | Reference  1.69 (1.15–2.01) | < 0.001 |
| Perineural invasion  Negative  Positive | Reference  2.00 (1.36–3.42) | 0.009 | Reference  2.32 (1.21–3.87) | 0.009 | Reference  2.10 (1.21–3.93) | 0.011 |
| Lymphatic invasion  Negative  Positive | Reference  2.57 (1.51–4.98) | 0.012 | Reference  2.50 (1.51–4.98) | 0.010 | Reference  2.43 (1.50–4.98) | 0.013 |
| Surgical approach  Abdominal  Thoracoabdominal | 1.70 (0.88, 2.60) | 0.078 | 1.72 (0.67, 2.87) | 0.094 | 1.82 (0.95, 2.98) | 0.087 |
| pTNM stage  I  II  III | Reference  2.43 (1.58–4.76)  6.14 (2.66, 8.67) | 0.008  0.003 | Reference  2.51 (1.48–4.66)  6.18 (2.88, 8.67) | 0.007  0.005 | Reference  2.51 (1.88–5.21)  6.04 (2.23, 8.12) | 0.009  0.003 |
| Adjuvant chemotherapy  No  Yes | Reference  1.08 (0.78, 1.38) | 0.231 | Reference  1.18 (0.80, 1.56) | 0.412 | Reference  1.10 (0.81, 1.51) | 0.323 |
| COUNT scores  Low (< 2)  High (≥ 3) | Reference  1.61 (1.11, 2.67) | 0.004 | Reference  1.60 (1.22, 2.67) | 0.008 | Reference  1.69 (1.15, 2.50) | 0.001 |
| mSIS  0  1  2 | Reference  1.83 (1.22, 2.78)  2.91 (1.32, 3.35) | < 0.001  < 0.001 | Reference  1.81 (1.28, 2.89)  2.80 (1.23, 3.21) | < 0.001  < 0.001 | Reference  1.92 (1.18, 2.98)  2.82 (1.10, 3.89) | < 0.001  < 0.001 |

**Supplemental Table 3.** Multivariate analysis of clinicopathologic variables in relation to OS in patients with AEJ. OS, overall survival. AEG, adenocarcinoma of the gastroesophageal junction. BMI, body mass index. ASA, American Society of Anesthesiologists. COUNT, controlling nutritional status. mSIS, modified systemic inflammation score.
