# Supplementary material for: Modified Systemic Inflammation Score Is an Independent Predictor of Long-Term Outcome in Patients Undergoing Surgery for Adenocarcinoma of the Esophagogastric Junction
Source: Front Surg. 2021 Nov 8;8:622821. doi: 10.3389/fsurg.2021.622821 (PMC8606684; doi:10.3389/fsurg.2021.622821)
Supplement: Supplementary Table 4 — Univariate and multivariate analysis of clinicopathologic variables in relation to RFS in patients with AEJ. RFS, relapse-free survival; AEG, adenocarcinoma of the gastroesophageal junction; BMI, body mass index; ASA, American Society of Anesthesiologists; COUNT, controlling nutritional status; mSIS, modified systemic inflammation score. [file Table_4.DOCX]

| Siewert classification | Type I | | Type II | | Type III | |
| --- | --- | --- | --- | --- | --- | --- |
| Clinicopathological features | HR (95% CI) | P value | HR (95% CI) | P value | HR (95% CI) | P value |
| Age | 1.21 (0.75, 2.87) | 0.315 | 1.68 (0.80, 2.71) | 0.146 | 1.40 (0.89, 2.02) | 0.101 |
| Gender  Male  Female | Reference  0.83 (0.57–1.99) | 0.221 | Reference  0.88 (0.64–1.89) | 0.312 | Reference  0.86 (0.60–1.78) | 0.365 |
| BMI | 1.26 (0.70, 2.43) | 0.208 | 1.22 (0.67, 2.45) | 0.198 | 1.31 (0.75, 2.49) | 0.237 |
| ASA score  1  2  3 | Reference  1.32 (0.62, 1.83)  1.20 (0.68, 2.12) | 0.312  0.212 | Reference  1.30 (0.61, 1.85)  1.22 (0.72, 2.16) | 0.302  0.215 | Reference  1.42 (0.58, 1.98)  1.26 (0.72, 2.36) | 0.310  0.228 |
| Tumor size (cm) | 1.59 (1.12, 2.45) | < 0.001 | 1.55 (1.10, 2.76) | < 0.001 | 1.54 (1.19, 2.45) | < 0.001 |
| Tumor differentiation  G1  G2  G3 | Reference  1.48 (1.21, 2.67)  1.86 (1.29, 3.58) | 0.010  0.003 | Reference  1.42 (1.22, 2.19)  1.92 (1.31, 3.89) | 0.006  0.003 | Reference  1.38 (1.19, 2.35)  2.01 (1.12, 3.77) | 0.008  0.009 |
| Vascular invasion  Negative  Positive | Reference  1.63 (1.22–2.18) | < 0.001 | Reference  1.60 (1.17–2.08) | < 0.001 | Reference  1.69 (1.22–2.16) | < 0.001 |
| Perineural invasion  Negative  Positive | Reference  2.09 (1.46–3.89) | 0.009 | Reference  2.01 (1.42–3.58) | 0.011 | Reference  1.98 (1.38–3.46) | 0.008 |
| Lymphatic invasion  Negative  Positive | Reference  2.48 (1.45–4.98) | 0.014 | Reference  2.56 (1.43–4.65) | 0.012 | Reference  2.85 (1.61–4.78) | 0.009 |
| Surgical approach  Abdominal  Thoracoabdominal | 1.72 (0.87, 2.61) | 0.089 | 1.79 (0.90, 2.62) | 0.081 | 1.81 (0.93, 2.67) | 0.098 |
| pTNM stage  I  II  III | Reference  2.42 (1.61–4.11)  6.16 (2.81, 8.97) | 0.001  0.012 | Reference  2.43 (1.63–4.31)  6.08 (2.71, 8.92) | 0.006  0.005 | Reference  2.51 (1.69–4.55)  6.09 (2.70, 8.90) | 0.009  0.002 |
| Adjuvant chemotherapy  No  Yes | Reference  1.11 (0.80, 1.42) | 0.319 | Reference  1.10 (0.82, 1.40) | 0.312 | Reference  1.15 (0.89, 1.52) | 0.322 |
| COUNT scores  Low (< 2)  High (≥ 3) | Reference  1.68 (1.22, 2.68) | 0.006 | Reference  1.71 (1.22, 2.67) | 0.008 | Reference  1.60 (1.12, 2.52) | 0.007 |
| mSIS  0  1  2 | Reference  1.89 (1.22, 2.71)  2.80 (1.31, 3.65) | < 0.001  < 0.001 | Reference  1.92 (1.18, 2.85)  2.67 (1.18, 3.45) | < 0.001  < 0.001 | Reference  1.76 (1.22, 2.79)  2.91 (1.35, 3.19) | < 0.001  < 0.001 |

**Supplemental Table 4.** multivariate analysis of clinicopathologic variables in relation to RFS in patients with AEJ. RFS, relapse-free survival. AEG, adenocarcinoma of the gastroesophageal junction. BMI, body mass index. ASA, American Society of Anesthesiologists. COUNT, controlling nutritional status. mSIS, modified systemic inflammation score.
